# Reinforcing the role of the conventional C-arm - a novel method for simplified distal interlocking

**DOI:** 10.1186/1471-2474-13-8

**Published:** 2012-01-25

**Authors:** Markus Windolf, Josh Schroeder, Ladina Fliri, Benno Dicht, Meir Liebergall, R Geoff Richards

**Affiliations:** 1 AO Research Institute Davos, Clavadelerstrasse 8, 7270 Davos, Switzerland; 2 Orthopedic department Hadassah Hebrew University Medical Center, Jerusalem, Israel; 3 Cardiff School of Biosciences, Cardiff University, Wales, UK

**Keywords:** Distal interlocking, Distal targeting, Nailing, Free-hand locking, Computer aided surgery

## Abstract

**Background:**

The common practice for insertion of distal locking screws of intramedullary nails is a freehand technique under fluoroscopic control. The process is technically demanding, time-consuming and afflicted to considerable radiation exposure of the patient and the surgical personnel. A new concept is introduced utilizing information from within conventional radiographic images to help accurately guide the surgeon to place the interlocking bolt into the interlocking hole. The newly developed technique was compared to conventional freehand in an operating room (OR) like setting on human cadaveric lower legs in terms of operating time and radiation exposure.

**Methods:**

The proposed concept (guided freehand), generally based on the freehand gold standard, additionally guides the surgeon by means of visible landmarks projected into the C-arm image. A computer program plans the correct drilling trajectory by processing the lens-shaped hole projections of the interlocking holes from a single image. Holes can be drilled by visually aligning the drill to the planned trajectory. Besides a conventional C-arm, no additional tracking or navigation equipment is required.

Ten fresh frozen human below-knee specimens were instrumented with an Expert Tibial Nail (Synthes GmbH, Switzerland). The implants were distally locked by performing the newly proposed technique as well as the conventional freehand technique on each specimen. An orthopedic resident surgeon inserted four distal screws per procedure. Operating time, number of images and radiation time were recorded and statistically compared between interlocking techniques using non-parametric tests.

**Results:**

A 58% reduction in number of taken images per screw was found for the guided freehand technique (7.4 ± 3.4) (mean ± SD) compared to the freehand technique (17.6 ± 10.3) (*p *< 0.001). Total radiation time (all 4 screws) was 55% lower for the guided freehand technique compared to conventional freehand (*p *= 0.001). Operating time per screw (from first shot to screw tightened) was on average 22% reduced by guided freehand (*p *= 0.018).

**Conclusions:**

In an experimental setting, the newly developed guided freehand technique for distal interlocking has proven to markedly reduce radiation exposure when compared to the conventional freehand technique. The method utilizes established clinical workflows and does not require cost intensive add-on devices or extensive training. The underlying principle carries potential to assist implant positioning in numerous other applications within orthopedics and trauma from screw insertions to placement of plates, nails or prostheses.

## Background

The gold standard for surgical treatment of diaphyseal lower limb long bone fractures is the use of intramedullary nails [1]. These nails are locked into place with proximal and distal screws to prevent rotation and shortening of the fractured limb. For proximal interlocking specialized jigs offer easy placement of the screws. However, due to deflection of the nail during insertion into the intramedullary canal no such automated process exists for the distal screws. The common practice for insertion of distal locking screws is a freehand technique under fluoroscopic control. The C-arm is tilted until alignment of interlocking hole axis and image intensifier beam is achieved indicated by a round appearance of the hole projected on the image. The drill is then manually oriented under repeated fluoroscopic control. The process is technically demanding and requires experience of the operator [2]. Depending on the skill level of the surgeon, procedure time and, even more important, radiation exposure to patient and surgical personnel can increase markedly [3]. In addition, a high rate of screw misplacements is reported [4].

A variety of approaches have been suggested to solve the distal locking problem. Recent advancements range from various mechanical aiming devices [5,6] continued to the idea of drilling from the inside to the outside (Dgimed Ortho. Inc., Minnetonka, US) over the use of surgical navigation systems [7-9] over laser illumination of the hole from the inside of the nail [4] to ultrasound [10] or electromagnetic hole locators [11,12]. Solutions appear to be technically irreproducible, are restricted to a specific nail/procedure or face the problem of extensive equipment requirements, which seems unjustified in the face of the comparatively simple problem of inserting a screw into a drill hole. Requirements for additional staff and long training periods with gradual learning curves lead many of these new ideas out of favor [13].

A new fluoroscopy based technique (guided freehand) was developed for simplified distal interlocking, aiming at reducing radiation exposure and operation time. The technique is generally based on the freehand standard and additionally guides the surgeon by means of visible landmarks projected on the C-arm image. A computer program plans the exact drilling trajectory by 2D-3D conversion of the locking hole projections from a single fluoroscopy shot in an arbitrary orientation and provides mentioned guiding landmarks in real-time on the familiar fluoroscopy screen. Interlocking holes can be drilled by visually aligning the drill to the planned trajectory. No additional tracking or navigation equipment is required.

Object tracking by utilizing X-ray projections of cylindrical holes carries potential for a variety of applications within trauma and orthopedics for positioning implants and instruments, such as screw insertions, guide-wire placements, positioning of plates, nails or prostheses was well as anatomical fracture reduction. The hereby introduced guided freehand distal locking system is considered as pilot application of the overall concept.

The aim of the study was to investigate this technique on human cadaveric specimens in an operating room (OR) environment, with realistic OR settings. The guided freehand procedure was compared with the conventional freehand technique in terms of radiation exposure and operational time.

## Methods

### Surgical technique

#### Guided freehand technique

**Figure 1:**
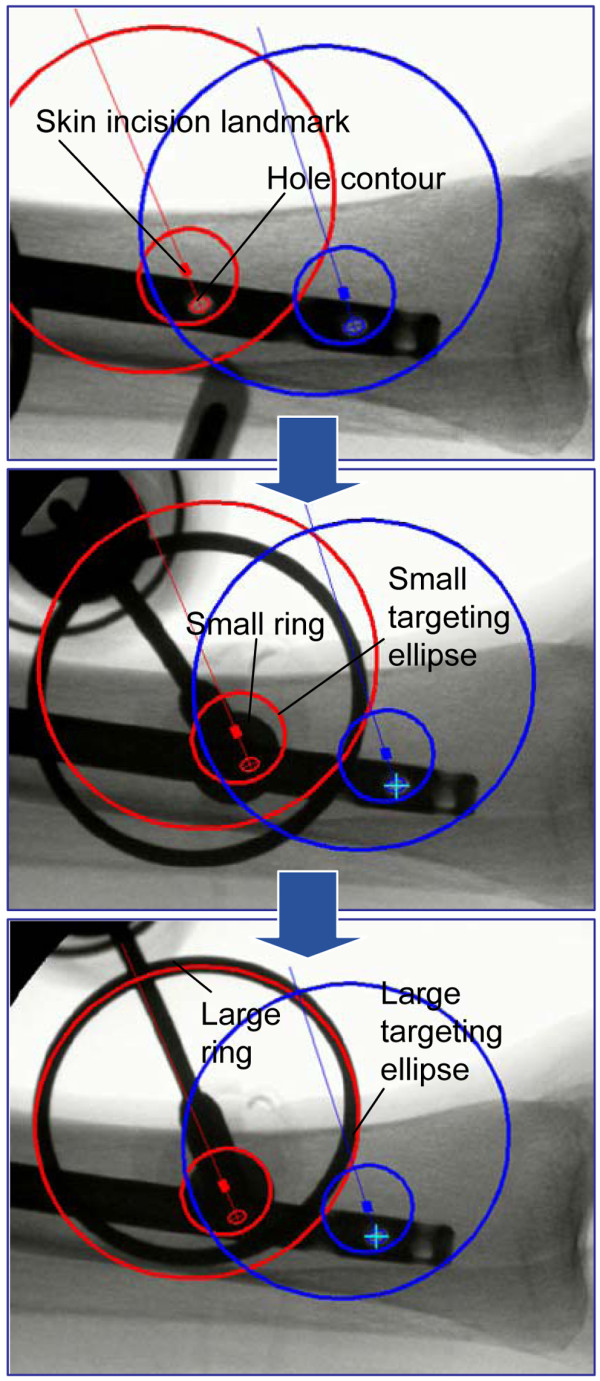
**Guided freehand procedure**. Top: A single image is taken from the distal part of the nail in arbitrary C-arm orientation. Guiding landmarks are calculated from the hole contours and projected into the image. A skin incision is performed at the incision landmark. Middle: Targeting jig and drill are inserted through the incision onto the bone surface. Under fluoroscopic control the jig is translated until the projection of the small ring coincides with the small targeting ellipse. Bottom: The jig is rotated until alignment of the projection of the large ring and the large targeting ellipse is achieved for drilling the hole

**Figure 2:**
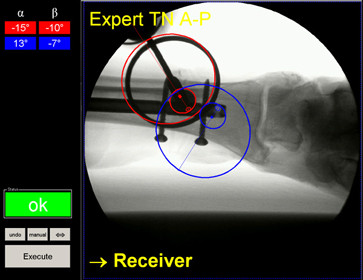
**Screenshot of the graphical user interface of the custom-made software algorithm**. A real-time window on the right displays the monitor signal of the C-arm with additional guiding landmarks. The left side of the window comprises the control elements to execute the calculation

**Figure 3:**
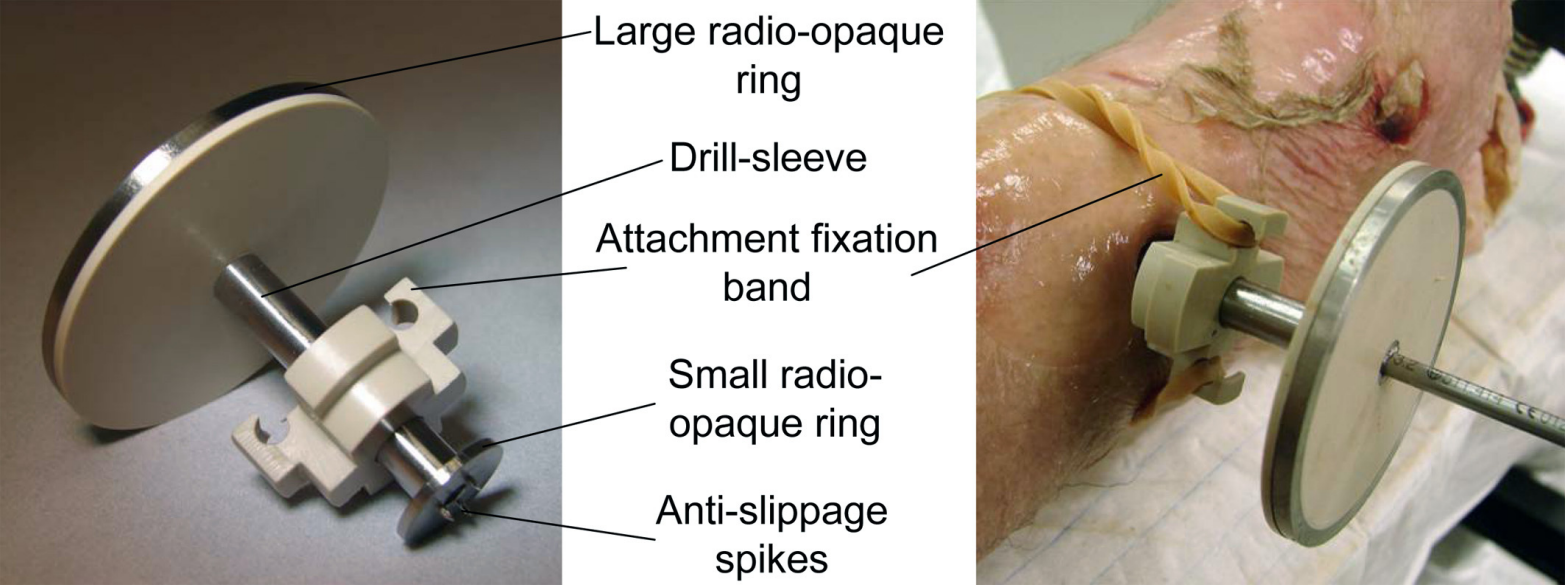
**Prototype of the targeting jig**. Two radio-opaque rings, concentrically arranged around a drill sleeve, are used as targeting elements within a fluoroscopic projection. The small ring is inserted through a skin incision onto the cortex of the bone. Here, a rubber band was used for stabilizing the jig during handling

One fluoroscopic shot of the distal tibia is taken in arbitrary orientation of the C-arm displaying two mediolateral or anteroposterior locking holes (Figure 1). No alignment between X-ray beam and interlocking hole axis is required. The C-arm can remain at its initial position. An external computer, equipped with an image acquisition device (here a VGA frame-grabber, Epiphan VGA2USB HR, Epiphan Systems Inc., Ottawa, Canada) is connected to the monitor output signal of the C-arm allowing real-time image processing. Said C-arm image is captured by the computer and processed with a custom-made software algorithm (Matlab, Mathworks Inc., Natick, USA) (Figure 2, Additional file 1: Video S1). The software is designed in a "one-button" fashion and requires less than 1 s processing time for two holes on a conventional computer. Internally the following computational steps are performed:

1. Automatic detection of two lens-shaped hole-projections from the image and extraction of significant landmarks from the hole contours.

2. Establishment of a virtual representation of the hole-cylinders and calculation of their virtual projection landmarks with respect to the cylinder orientation.

3. Determination of the spatial orientation of both hole-cylinders in 6° of freedom (DOF) by matching the virtual- with the actual projection landmarks by numerical optimization.

4. Virtually aligning a representation of a targeting jig with the calculated hole-axes. The jig comprises a drill sleeve and two radio-opaque rings (one large and one small) concentrically arranged around the sleeve (Figure 3).

5. Projecting two targeting circles (corresponding to the radio-opaque rings) and a skin incision point for each drill hole into the C-arm image (Figure 1).

6. Routing the image back to the monitor of the C-arm.

After computation, the targeting elements are visualized on the image intensifier. Under fluoroscopic control a 2 cm skin incision is created with a scalpel at the position of the incision landmark. The targeting jig is placed over the drill bit and is inserted through the incision onto the bone surface. Under fluoroscopic control, the jig is translated on the cortex by moving the drill until the projection of said small ring coincides with the small targeting ellipse (Figure 1). Spikes at the targeting jig prevent slippage of the drill bit (Figure 3). Subsequently the drill is tilted until the projection of the large ring coincides with the large targeting ellipse. The achieved orientation allows drilling of the hole and subsequent insertion of a screw. The procedure is then repeated for the second interlocking hole. During the process the C-arm can be maintained at its initial position. No pivoting of the C-arm to achieve circular hole projections is required. The order of processing the interlocking holes can be freely chosen. The general procedure is illustrated in a workbench fashion on a separated plastic tibia in Additional file 2: Video S2.

#### Freehand

Lens-shaped hole projections indicate misalignment between the interlocking hole axis and the image intensifier beam. The C-arm is tilted by adjusting the two cardanic angles under repeated fluoroscopic control until the screw hole appears round on the radiographic projection. In a second stage, the tip of the drill bit is placed in the center of the projected screw hole. Subsequently, the drill is visually oriented to the central axis of the C-arm and the drill-hole is created. All steps are performed under repeated fluoroscopic control.

### Experiment

#### Setup

**Figure 4:**
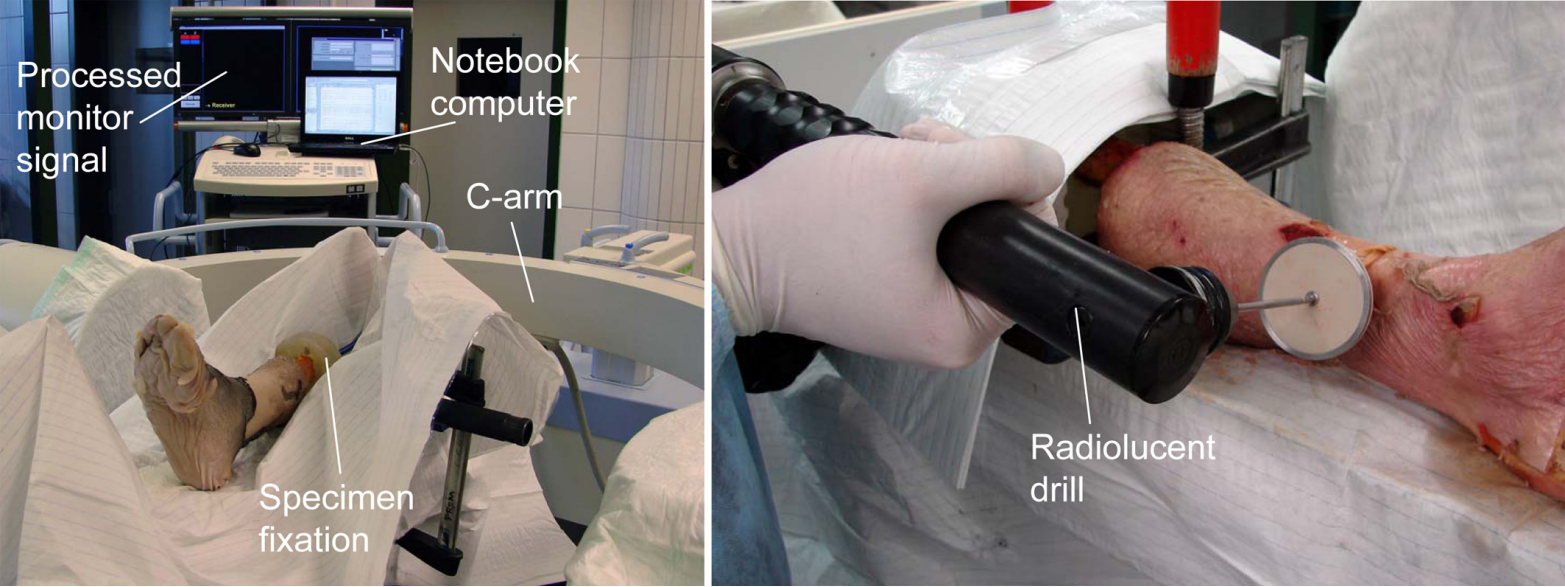
**Experimental setup**. Left: Cadaveric below knee specimens, instrumented with a tibia nail, were secured to an OR table. A conventional 2D C-arm could be freely positioned to visualize the distal interlocking holes. The C-arm monitor signal was processed in real-time and was routed back to the C-arm monitor. Right: A radiolucent drill was manually operated either with aid of a targeting jig or without

Ten fresh frozen human below-knee specimens (five left, five right) were used with appropriate consent of the relatives. Specimens were already part of a different study and reused for the present investigation. Intact bone structure and soft tissue at the distal part of the tibia were ensured. All specimens were long enough to provide proper placement and anchoring of the nail. The proximal ends of the tibia and fibula were cylindrically embedded in Polymethylmethacrylate (Beracryl, Suter Kunststoffe AG, Fraubrunnen, Switzerland) in order to mount the specimens to the OR table. According to the size of the bones, a 9-11 mm diameter Titanium Expert Tibia Nail (Synthes GmbH, Bettlach, Switzerland) was inserted into the intramedullary channel of the tibia in a non-standardized manner to avoid predictable orientation of the distal locking holes inside the bone. The nail was proximally clamped to the embedding with a screw to prevent nail movement. In order to provide an experimental setting close to surgical practice, the locking procedures were performed in a standard operating room with optimal lighting and generalized conditions (Figure 4). In order to mimic a real life lower limb, the hindfoot rested distally on the OR table. Proximally the specimens were clamped with a vise fixed to the table. The limbs were draped in standard surgical cloths covering the protruding proximal end of the nail to conceal its orientation. A Siemens ARCADIS Varic C-arm system (Siemens AG, Munich, Germany) was used as imaging means for all procedures. The C-arm recorded 1024 × 1024 pixel gray-value images. Automatic intensity correction was enabled. All locking procedures were performed by a single fourth year orthopedic resident surgeon, who had experienced about 30 procedures of distal locking in clinics prior to this study. The surgeon was dressed in full operating room gear including protective glasses, a lead dressing gown, surgical robes and double gloves. A single C-arm technician operated the C-arm according to the surgeon's commands. A battery powered radiolucent drive (511.300, Synthes GmbH, Bettlach, Switzerland) with a Ø 3.2 mm (length 148/122 mm) standard drill bit was used for drilling. Conventional Ø 3.9 mm (length 40 mm) Titanium self-tapping locking bolts were inserted with a hexagon screw driver.

#### Procedure

For each interlocking procedure all four distal locking holes of the Expert Tibia Nail were filled with screws (2 mediolateral (ML) and 2 anteroposterior (AP)). Each specimen was operated twice by retrieving the nail 4-5 cm after the first procedure to account for anatomical differences along the bone axis. Both interlocking methods (freehand/guided freehand) were performed on the specimens in random order. Prior to each interlocking procedure the C-arm was retracted from the OR table and the C-arm tilt was reset. After the screw was inserted, a control radiograph was taken to exclude misplacement. In case of a screw missing the interlocking hole, the procedure was continued until the screw penetrated the hole.

#### Data acquisition and evaluation

Net operation time was measured from the first C-arm shot until screw was tightened for each interlocking hole. Number of fluoroscopy shots and radiation time were recorded for each interlocking hole. The control shot was included in the evaluation for both techniques. After assessing data distribution (Shapiro-Wilk test) non-parametric test statistics (Mann-Whitney-*U *test) were carried out to identify differences between the two interlocking techniques regarding operating time and radiation exposure. The significance level was set to α = 0.05.

## Results

A total of 18 complete interlocking procedures (4 screws each) were performed on 10 specimens. 2 pilot tests were excluded from the analysis. 8 valid procedures were performed with the freehand technique and 10 procedures were carried out with the guided freehand technique. There were two misplaced screws with the freehand technique whereas with guided freehand all screws were inserted correctly.

### Radiation

Average number of fluoroscopy shots per inserted screw was 7.4 ± 3.4 (mean ± standard deviation) for the guided freehand technique and 17.6 ± 10.3 for the freehand technique, corresponding to a reduction of 58% when using guided freehand (Table 1) (*p *< 0.001). When considering only the mediolateral screws a 59% reduction in average number of fluoroscopy shots per inserted screw was found (*p *< 0.001). Total radiation time for all 4 screws was reduced by 55% when using guided freehand (17.1 ± 3.7 s) compared to the freehand technique (37.9 ± 9.1 s) (*p *= 0.001).

**Table 1:** Experimental results

		Number of radiographs			Radiation time [s]			Operation time [min]	
	**Freehand**	**Guided Freehand**	**Red. [%]**	**Freehand**	**Guided Freehand**	**Red. [%]**	**Freehand**	**Guided Freehand**	**Red. [%]**

**2 ML screws**	14.3 ± 5.6	5.9 ± 2.6	59	8.3 ± 3.2	3.4 ± 1.5	59	3.6 ± 1.2	2.7 ± 0.9	25

**2 AP screws**	21.7 ± 13.2	8.9 ± 3.6	59	12.6 ± 7.7	5.2 ± 2.1	59	4.8 ± 2.8	3.4 ± 1.2	29

**per inserted screw**	17.6 ± 10.3	7.4 ± 3.4	58	10.2 ± 6.0	4.3 ± 2.0	58	4.1 ± 2.1	3.2 ± 1.2	22

**per procedure (4 screws)**	65.3 ± 15.7	29.5 ± 6.4	55	37.9 ± 9.1	17.1 ± 3.7	55	15.5 ± 4.2	12.7 ± 2.9	18

Number of taken radiographs, radiation time and operation time for the conventional freehand technique as well as for the proposed guided freehand method. Numbers represent mean ± standard deviation and percentage of reduction due to guided freehand

### Operation time

With 4.1 ± 2.1 min per screw for the freehand technique and 3.2 ± 1.2 min for the guided freehand technique, a significant reduction of 22% in operation was achieved when using guided freehand (*p *= 0.018). When considering only the two mediolateral screws, the time reduction accounted to 25% with guided freehand (*p *= 0.013).

Results are summarized in Table 1.

## Discussion

Distal locking of intramedullary nails is a frequently employed, but technically demanding procedure in trauma surgery. In this paper a newly developed technique for implant independent distal interlocking (guided freehand) is introduced. The method was compared to the common freehand technique in an experimental cadaveric setting exemplified on conventional tibia nails. The guided freehand technique has proven to reduce radiation exposure by more than 50% and operation time by 20% when compared to freehand.

When a nail is inserted into a long bone, it is likely to bend according to the curvature of the intramedullary canal [14]. Exact orientation of the distal interlocking holes is, hence, difficult to predict. Usually surgeons use repeated fluoroscopy to insert the screws in a freehand manner. Radiation is a growing problem amongst orthopedic surgeons, associated with a relative risk for cancer of 5.37 with respect to the general population [15]. Malignancies of exposed personnel range from cancers of solid organs (i.e. thyroid and pancreas), to skin and hematopoietic cancers [16]. In female orthopedic surgeons the standardized prevalence ratio for all cancers is 1.9 and 2.88 specifically for breast cancer when compared to the general population [17]. In the literature radiation expenditure for freehand locking differs widely. E.g. Gugala et al. reported a fluoroscopy time of 36 s for placement of two screws in the tibia [3], whereas Suhm et al. stated intense use of fluoroscopy during freehand locking of 108 s per screw [9]. Factors such as experience level of the operator or experimental/clinical setup might contribute to this scattering. In this study all screws were placed by a single 4th year orthopedic resident to test the system on a young experienced surgeon. Recorded average radiation time during conventional freehand locking was 35 s for two locking screws, which is in the lower range of the reported values. In the investigation of Kirousis et al. [2] a complete tibia nailing procedure required 72 s of radiation and resulted in an effective dose of 0.04 mSv for the operating surgeon and 0.11 mSv for the C-arm technician. In view of the annual dose constraint of 10 mSv (International Commission on Radiological Protection [18]) efficient and responsible use of radiation is of utmost importance.

Opposed to the mentioned disadvantages of freehand locking such as radiation exposure and handling complexity, marginal requirements for recourses and equipment make the technique practicable all over the globe. Guided freehand does not aim to replace but to enhance the freehand gold standard. Tilting of the C-arm to achieve round hole-projections implies 3D vision of the surgeon and often generates the major portion of time and radiation. Some operators rotate the patient rather than the C-arm, which could end in loss of fracture reduction and anatomical mal-rotation. With guided freehand the C-arm can be maintained at a convenient position. The demanding step of aligning the drill to the image intensifier projection axis is replaced by intuitive matching of targeting structures, easy to follow by the inexperienced surgeon.

A variety of options for improved distal interlocking has been proposed in the past decades. None has yet found its way into accepted clinical practice. With fluoroscopy based navigation for distal interlocking an impressive reduction in radiation could be reported (2 s radiation per screw). However, the authors state that fluoroscopy based surgical navigation markedly increases the need for resources [19]. Up to an additional 40 min were required prior to skin incision and after skin closure as set up and take down time for the navigation system. Moreover, an especially trained technician was needed [20]. Ultrasound based techniques using differences in resonance between nail and bone [10] lack accuracy. Electromagnetic solutions offer acceptable accuracy without direct involvement of radiation. However, radiographic imaging is still required to confirm proper screw position at the end. Electromagnetic tracking implies the insertion of a pre-calibrated single-use probe into the cannulation of the nail with a cable connection to the outside [12,21]. Besides additional bridging between sterile and non-sterile fields, the blocked cannulation restricts the surgeon to a specific sequence of screw insertion progressing from distal to proximal, which is contradictory to clinical practice. Moreover, all magnetic metals such as stainless steel implants, screwdrivers or drill-bits cannot be used. It is a point of discussion whether it is worth interrupting the clinical workflow and setting up of an additional support system with considerable restrictions for the sole task of distal interlocking.

The hereby proposed concept is consequently geared to existing surgical workflows. Besides a simple targeting jig and software, no additional equipment is required. A conventional C-arm, as available in the bulk of operation theaters worldwide, is essential for nailing. It appears, therefore, reasonable to further involve the C-arm for distal interlocking when it is already employed for nail insertion, fracture reduction and implant positioning beforehand. The authors believe that the potential of conventional radiographic devices is only barely taped since the intended use is confined to plain visualization. Elevating the role of the conventional C-arm from pure imaging could significantly contribute to an improved surgical outcome. Hidden information within 2D projections could be more efficiently extracted by innovative algorithms to aid the surgeon in the daily routine. The idea of reconstructing 3D information from 2D projections is certainly not a novelty. Cone beam algorithms, for example, build the basis for 3D reconstruction by computed tomography [22]. Some work has already been done on utilizing the image intensifier projections of distal locking holes to identify their orientation [23,24]. However, solutions appear clinically infeasible. At least two projections are required from different angles, computational time is long and no simple procedure has been suggested to finally position a tool for drilling.

The hereby proposed method assists in planning (identification of the hole orientation) and navigation of the drill by means of a 2D C-arm. The 3D transformation problem is solved from a single projection of two interlocking holes. No tilting of the C-arm is required to obtain a second view angle. Computational time for two holes is kept below 1 s and robustness is increased by extracting only significant landmarks from the hole contour rather than processing the entire projection shape. The technique is independent from a specific implant type or brand and could be universally applied to tibia, femur, humerus or to other relevant regions.

However, some issues need to be addressed in the future. As the algorithm processes two interlocking holes simultaneously, recalculation due to movement of the patient might be difficult after the first screw is already inserted. Currently, the prototype system simulates the second hole on basis of the previous calculation. Furthermore, patient movements relative to the C-arm can have adverse effects on the procedure. With the next X-ray the algorithm identifies and corrects these motion artifacts by real-time monitoring of shifts between images and performs automated recalculations. Nonetheless, the nature of the method remains static. Events occurring between distinct snapshots cannot be detected. The advantages of a freehand procedure are essentially to be seen in maximized freedom and usability for all kinds of applications independent from specific devices and implant families. However, from a handling perspective a freehand procedure remains demanding. In the present case, the skill level of the operator determines the extent of radiation needed for iterative control. For example a handheld drill-bit happens to slip on the cortex requiring restart of targeting. Slippage and soft tissues wrapping around the drill-bit could be improved by the proposed targeting jig. Still, the screw is freely inserted into the drill-hole. Particularly in reduced bone quality, mal-placement of the screw might occur even if the drill channel was correct.

Abstracting the underlying idea as a future outlook, an intramedullary nail with interlocking holes can be regarded as a radio-opaque object with two cylindrical holes, which can be spatially tracked from 2D projections. Holes could therefore be regarded as tracking markers. Even though, all kinds of geometries are thinkable to serve as markers, cylindrical holes appear favorable because they are easy to produce (or already existent) and their projection is explicit to track. It is therefore credible to establish a support system for surgical routine interventions, e.g. for controlling the entire nailing procedure from implant positioning to distal interlocking.

## Conclusions

A new approach for improved distal locking of intramedullary nails is proposed based on image processing of conventional X-rays. In an experimental setting, the "guided freehand" technique reduces radiation exposure by more than 50% and operation time by 20% compared to freehand locking without the need for expensive equipment or extensive training. The concept carries potential for assisting implant positioning in various fields within orthopedics and trauma.

## Competing interests

The authors are not compensated and there are no other institutional subsidies, corporate affiliations, or funding sources supporting this work unless clearly documented and disclosed.

## Authors' contributions

MW developed the concept, programmed the software algorithms and drafted the manuscript. JS performed the surgeries and drafted the manuscript. LF planned and supervised the experiments, evaluated the data and helped in writing the manuscript. BD designed and produced the prototype targeting jig. ML contributed to study planning and supported the paper draft. GR supported study planning and the concept development process and revised the manuscript. All authors read and approved the final manuscript.

## Pre-publication history

The pre-publication history for this paper can be accessed here:

http://www.biomedcentral.com/1471-2474/13/8/prepub

## Supplementary Material

Additional file 1 **Video S1**. Click here for file

Additional file 2 **Video S2**. Click here for file
